# Protective role of fish oil (Maxepa) on early events of rat mammary carcinogenesis by modulation of DNA-protein crosslinks, cell proliferation and p53 expression

**DOI:** 10.1186/1475-2867-7-6

**Published:** 2007-05-01

**Authors:** Sangita Manna, Tridib Chakraborty, Suresh Damodaran, Kartick Samanta, Basabi Rana, Malay Chatterjee

**Affiliations:** 1 Division of Biochemistry, Department of Pharmaceutical Technology, Jadavpur University, P.O. Box – 17028, Kolkata-700032, India; 2 Cardiovascular and Cancer Research Institute, Texas A&M University System, Health Sciences Center, College of Medicine, Temple, TX-76504, USA

## Abstract

**Background:**

Fish oil is known to protect from many types of cancers of the colon, liver, breast, prostate and lung [1-3]. The objective of the present study was to evaluate the role of fish oil [Maxepa, supplemented at a dose of 0.5 ml is equivalent to 90 mg eicosapentaenoic acid (EPA) and 60 mg docosahexaenoic acid (DHA)] on cell proliferation, expression of p53 tumor suppressor protein and DNA protein crosslinks (DPCs) in a defined model of chemical rat mammary carcinogenesis. Mammary carcinogenesis was initiated by a single, intravenous (i.v.) tail vein injection of 7,12 dimethylbenz(α)anthracene (DMBA) at a dose of 5 mg DMBA/2 ml corn oil/kg body weight in female Sprague-Dawley rats at 7 weeks of age. Fish oil supplementation was started daily, 2 weeks prior to DMBA injection and continued for 24 (31 weeks of animal age) weeks and 35 (42 weeks of animal age) weeks of post DMBA injection, for histopathological and immunohistochemical and for morphological studies, respectively.

**Results:**

Our results indicate the chemopreventive effect of fish oil (Maxepa) on DMBA-induced rat mammary carcinogenesis. Administration of fish oil further showed a prominent reduction of cell proliferation (24.34%, P = 0.001); DPCs (25%, P < 0.001) and an increased expression of p53 protein (4.636 ± 0.19, P < 0.001) in preneoplastic mammary tissue when compared to carcinogen control counterpart. Histopathological and morphological analyses were carried out as end-point biomarkers.

**Conclusion:**

Our study thus provides evidence for the anticarcinogenic effect of fish oil (Maxepa) in limiting mammary preneoplasia in Sprague-Dawley rats.

## Background

Emerging evidences from epidemiological and experimental studies indicate a relationship between dietary fat and the risk of cancer [1-4]. Especially it has been shown that populations that consume high amounts of omega-3 fatty acids have lower incidence of breast, prostate and colon cancers than those who consume fewer amounts of n-3 fatty acids (5). It is also reported that dietary fish oil reduces the growth of subcutaneously transplanted R3230AC mammary adenocarcinoma cells in female F344 rats [6].

7,12 dimethylbenz(α)anthracene-(DMBA) induced mammary carcinogenesis in rats has been widely used in various mammary cancer chemopreventive studies [7,8]. This model appears to be most relevant to the development of human breast cancer, especially in its origin. In this model tumours appear from ductal epithelial cells as in most human breast cancers. Moreover, histogenesis, morphology and progression of hyperplastic premalignant and malignant lesions are similar in many aspects to those of human breast cancer [9].

Histopathology of a particular tissue is the basic marker for the study of neoplasia. DNA-protein crosslinks (DPCs) are proposed as indicators of early biological effects due to carcinogen exposure [10,11]. The isolation of DPCs is important not only for elucidation of functional and structural aspects of DNA protein interactions but also important for evaluation of genotoxicity of carcinogens. DPC is a type of DNA damage that could be used as biomarker for testing early carcinogenic exposure or early lesions of carcinogenic process [12]. Cell proliferation can influence carcinogenesis by a number of mechanisms [13]. The p53 tumor suppressor gene product appears to play a central role in carcinogenesis, and is integral in cell-cycle control, DNA repair, cellular differentiation and apoptosis [14]. The elevated expression of p53 can inhibit cell proliferation.

In this present study, we have investigated the anticarcinogenic role of fish oil (Maxepa) in a defined model of chemical rat mammary carcinogenesis. This might throw some light in understanding the role of these fatty acids in the inhibition of mammary carcinogenesis.

## Results

### General observation

During the entire term of the study, no treatment related alteration in the daily intake of food and drinking water was observed among the different groups of rats. No difference in behavior patterns like irritability, aggressiveness and apparent docility was noted.

### Mammary histology

**Figure 1:**
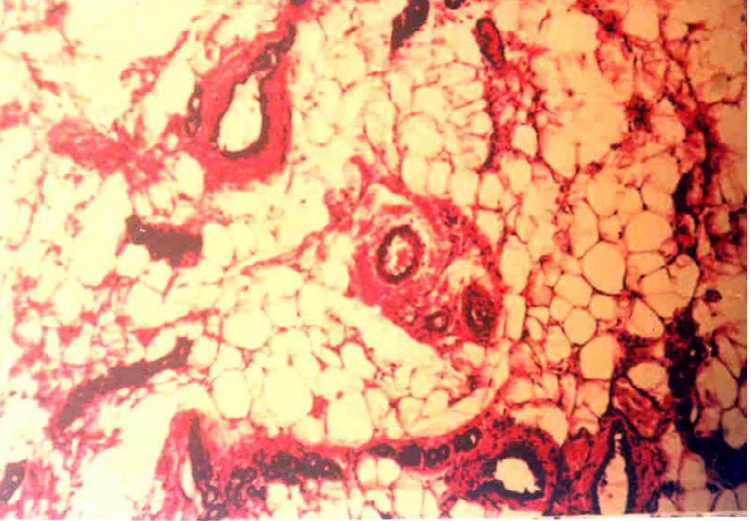
Histological section of mammary tissue of rats, showing normal cellular architecture (Normal Control, group A). Magnification, H & E × 40.

**Figure 2:**
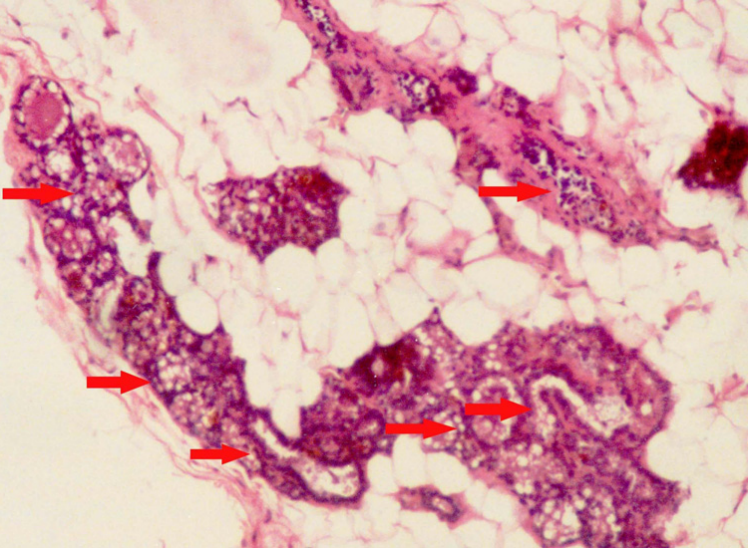
Histological section of mammary tissue of rats, showing marked proliferation of ductal epithelial lining with hyperchromatic enlarged nuclei (DMBA Control, group B) (marked with arrows). Magnification, H & E × 40.

**Figure 3:**
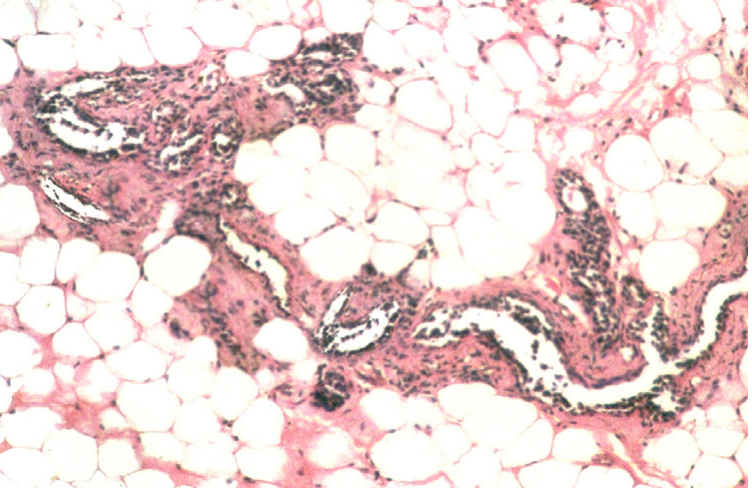
Histological section of mammary tissue of rats, showing almost normal cellular architecture with no signs of proliferation (DMBA + Maxepa Treatment, group C). Magnification, H & E × 40.

Figure 1, the normal control (group A) preserved normal ductular and alveolar structure of mammary tissue with epithelial cells of uniform appearance. Figure 2, mammary tissue from DMBA-treated group (group B) showed hyperplasia of the ductular cells characterized by multi-layering and clustering of the cell with nuclear hyper chromasia and pleomorphism. The layering went up to more than three cells and there were cellular bud formation in some places. The cellular architecture was found to be altered and enlargement of the alveolus was seen along with cells showing nuclear enlargement, clumping of chromatids and prominent nucleoli. It was observed from histological examination that the DMBA + fish oil-treated group (group C) presented a histological profile similar to the normal control (Fig. 3). Tissue from this group (group C) showed distinct reduction of hyperplasia as evidenced by only normal double layer of cells with regular round nuclei with presence of chromatin. The fish oil control group (group D) showed no observable distinct change from the normal control (figure not shown).

### Morphological findings

**Figure 4:**
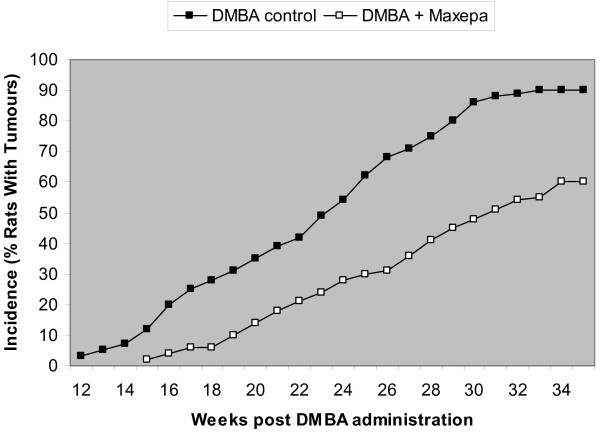
Cumulative incidence of mammary tumours (adenocarcinomas) in DMBA control (group B) as well as DMBA + Maxepa-treated (group C) groups on and from 10 weeks upto 35 weeks. P < 0.05 when compared to the DMBA control (group B) by Fisher's exact probability test.

The incidence (%) of palpable mammary tumours in the DMBA control (group B) and fish oil-supplemented DMBA group (group C) are shown in figure 4 and table 2. In the DMBA control group, 90% of animals had tumours at the end of 35 weeks (Table 2). The mean latency period of tumour appearance was as early as 12.1 ± 0.3 weeks post-carcinogen treatment. The mammary tumour usually appeared one at a time with additional tumours appearing even several weeks after the first tumour was observed. The majority of tumours appeared between 15 and 25 weeks and multiple tumours of different sizes were frequently found in the same animal. Most of the tumours were between 2 and 5 mm^3 ^in size (Table 2). Fish oil when given to the carcinogen-treated animals (group C), its inhibitory action could be ascertained from the reduced tumour incidence (Fig. 4 and Table 2). In this group only 60% of rats had tumours at the end of 35 weeks, which is significantly different (P < 0.05) from group B (Table 2). The mean latency period for tumour appearance for this group was 15.7 ± 0.5 weeks post-carcinogen treatment, which is significantly (P < 0.001) longer than group B. From figure 4, it can be observed that there was a decrease in the percentage of rats developing tumours with time in the fish oil treated group. Between 25 and 31 weeks after DMBA administration, significant (P < 0.001) lowering of the percentage of rats developing tumours were observed and again at a later stage (between 32 and 35 weeks), there was a significant (P < 0.05) reduction in tumour-incidence when compared to the carcinogen control group (Fig. 4). Fish oil treatment also characteristically attenuated the number of tumours of 2 and 5 mm^3 ^in size indicating slower tumour progression (Table 2).

**Table 2:** Effect of fish oil on DMBA-induced palpable mammary tumors in rats after 35 weeks

**Group**	**No. of rats with tumors^a ^per total rats**	**Tumor incidence (%)**	**Total no. of tumors**	**Tumor**	**Size (mm^3^)**
				**<2**	**>2–<5**

**B**	9/10	90	61	22	39
**C**	6/10	60^b^	32^c^	11	21

^a^Palpable tumors confirmed as adenocarcinomas by histopathology.

^b^Significantly different from group B by Fischer's exact probability test (P < 0.05).

^c^Significantly different from group B by Student's t-test (P < 0.01).

### Effect of Fish oil on induction of DPCs in vivo

**Figure 5:**
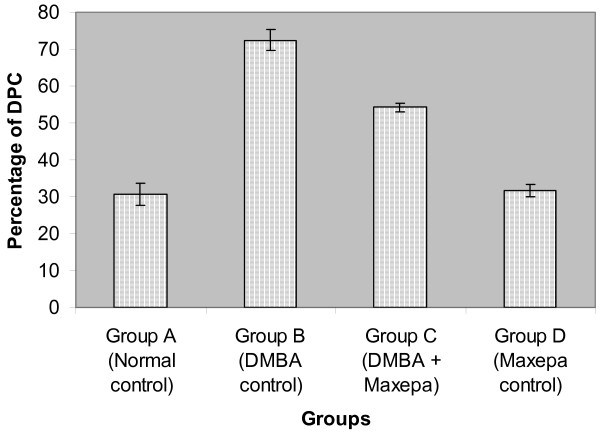
Effect of fish oil on the percentage of DNA-protein crosslinks (DPCs) in mammary tissue. Each column and bar represents Mean ± SEM. P < 0.001 when DMBA control (group B) is compared to normal control (group A). P < 0.001 when DMBA + Maxepa treated group (group C) is compared to DMBA control (group B). P < 0.001 when DMBA control (group B) is compared to Maxepa control (group C).

DMBA-treated group results a clear demonstration of protein associated with DNA breaks. The carcinogen control group exhibited an increase in DPC compared to their normal counterparts (P < 0.001). Treatment with fish oil (Maxepa) reduced the percentage of DPC by 25% (P < 0.001) when compared to carcinogen control counterparts. Thus, early changes during the carcinogenic process were associated with increased percentage of DPC that was reduced by treatment with fish oil (Fig. 5). Results were analyzed by Student's t-test and one-way ANOVA (F = 65535, F crit = 3.37, P < 0.001).

### Cell proliferation

**Figure 6:**
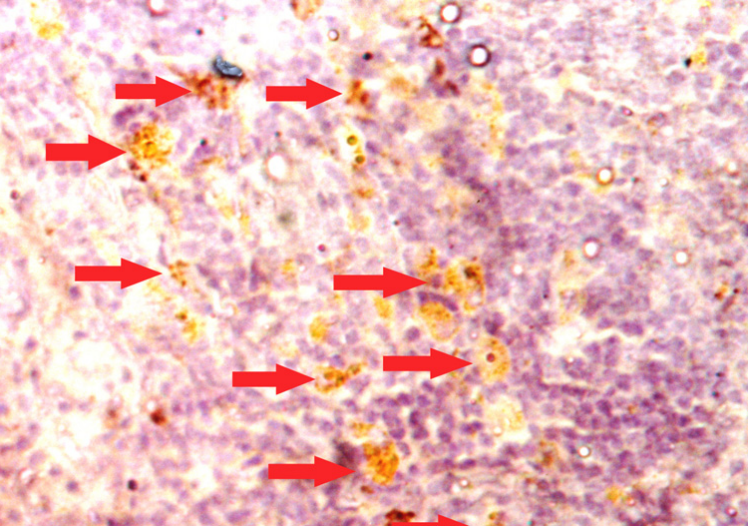
Representative immunohistochemistry photomicrograph of BrdU-labeling of rat mammary tissue [DMBA Control (group B)]. Arrows (→) indicate BrdU-labelled brown-stained cells. Magnification, × 40.

**Figure 7:**
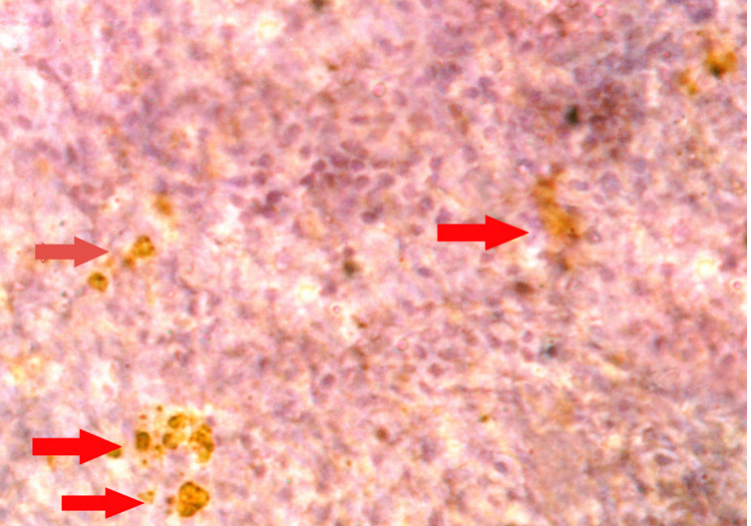
Representative immunohistochemistry photomicrograph of BrdU-labeling of rat mammary tissue [DMBA + Maxepa-treated group (group C)]. Arrows (→) indicate BrdU-labelled brown-stained cells. Magnification, × 40.

To analyze the chemopreventive action of fish oil (Maxepa), we examined its effect on cellular proliferation in the mammary gland. The percentage of BrdU-labelled cells as calculated by LI (labeling index) was more in the carcinogen control group B (Table 3). The BrdU-LI in the DMBA control group (Group B) was 26.7 ± 2.54 and in DMBA + fish oil group (Group C) was 20.2 ± 1.58. Thus, there was substantial reduction in BrdU-LI by 24.34%, (P = 0.001). The BrdU-labeled cells showed a distinct nuclear localization. Figures 6 and 7 indicate representative pictures of mammary tissue from the DMBA control group (group B) and the DMBA + fish oil-treated group (group C), respectively.

**Table 3:** BrdU-LI in mammary tissue

Groups	Brdu-LI^a^
Group B (DMBA control)	26.7 ± 2.54^b^
Group C (DMBA + Maxepa)	20.2 ± 1.58

LI = labeling index

BrdU-LI = percentage of BrdU-labeled cell/total number of cells counted.

^a^Approximately 200 cells were counted per field, 10 fields were examined per slide and 10 slides were examined per group.

^b^Values represents mean ± SEM.

### p53 immunoexpression

**Figure 8:**
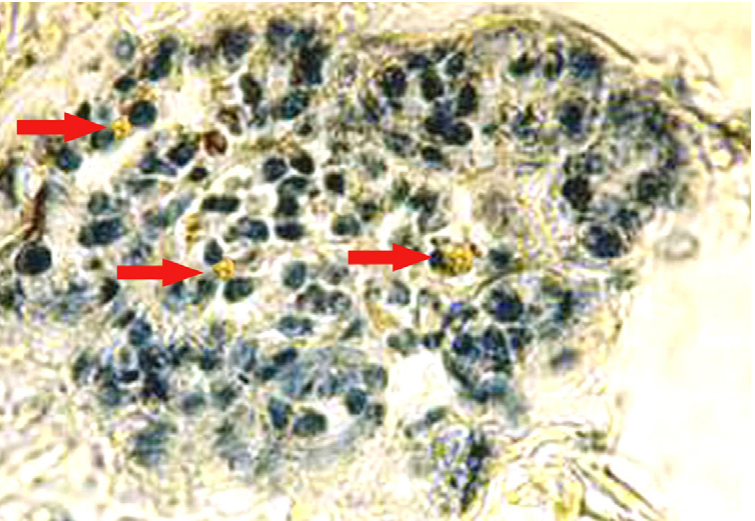
Immunolocalization of p53 in the mammary tissue of rats [DMBA Control (group B)] with anti-sheep p53 antibody (1:200) and AEC. Arrows (→) indicate p53 immuno-positive cells in mammary tissue of rats. Magnification, × 40.

**Figure 9:**
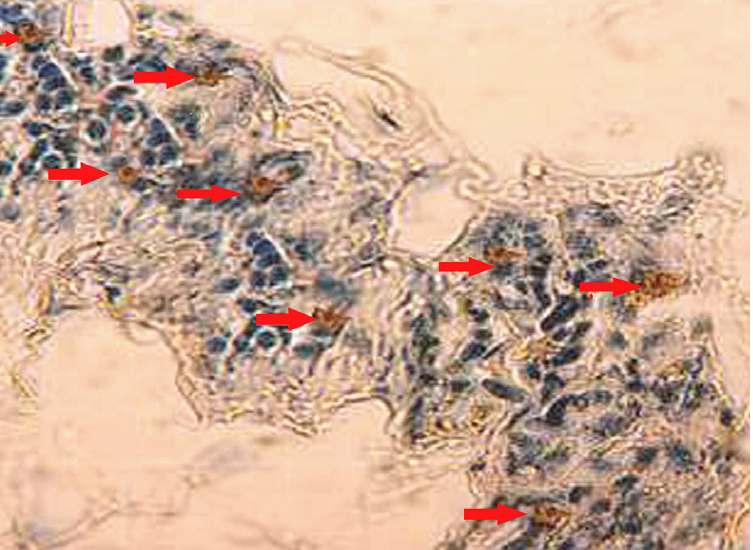
Immunolocalization of p53 in the mammary tissue of rats [DMBA + Maxepa-treated group (group C)] with anti-sheep p53 antibody (1:200) and AEC. Arrows (→) indicate p53 immuno-positive cells in mammary tissue of rats. Magnification, × 40.

A few p53 immuno-positive cells (1.484 ± 0.021) was detected in the mammary tissue sections of the carcinogen control group (group B) (Fig. 8) when compared to group A; whereas figure 9 showed an increase in p53 immunopositivity (4.636 ± 0.19, P < 0.001) upon fish oil supplementation (group C) when compared to DMBA control (group B). p53 expression in the normal control (group A) and Maxepa control (group D) were 8.456 ± 0.17 and 8.47 ± 0.16, respectively when compared to group B. One-way ANOVA showed significant changes in p53 expression among various groups of animals (F = 65535, F crit = 3.37, P < 0.001).

## Discussion

The present study highlights that fish oil (Maxepa) offers considerable protection against DMBA-induced mammary carcinogenesis in female Sprague-Dawley rats. Exposure to DMBA resulted in hyperplasia that was reverted almost near to normal following treatment with fish oil, as evidenced by detailed histopathological analysis, indicating the apparent chemopreventive potential of this oil. As there is a close relationship between hyperplasia and the subsequent steps leading to malignancy, the protective role of fish oil against mammary carcinogenesis may be manifested by gradual slowing of the occurrence of hyperplasia to a considerable extent and thereby delaying the appearance of tumour with the increased latency period [15,16].

Essential fatty acids are required for the normal structure of the membranes including cell membranes, mitochondrial membranes and nuclear membranes. They are the precursors of a whole series of second messengers [17] thereby regulating of the formation of cytokines, and modulate the behavior of most membrane bound proteins including receptors, ion channels and ATPase [18].

n-3 fatty acids may be found in vegetable oils, especially canola and soybean oil, and in green leafy vegetables as α-linolenic acid (LNA) [18:3 (n-3)] and in larger amounts in fatty cold water fish as eicosapentaenoic acid (EPA) [20:5 (n-3)] or docosahexaenoic acid (DHA) [22:6 (n-3)] [5]. n-3 PUFA predominate in chloroplasts of green plants (linolenic acid) and in phytoplanktons (EPA and DHA). Via the marine food chain, all forms of marine life become enriched with PUFA of the n-3 family [19].

An inhibitory role of fish oil was clearly indicated by the reduced incidence of DMBA induced mammary tumours in rats. An observation that a significant percentage of rats having less occurrence of tumours after fish oil treatment can be explained by assuming the carcinogenic effect, even if initiated, was suppressed to a considerable extent as indicated by a long latency period of tumour development [20]. The observed beneficial effect of fish oil on tumour growth as evidenced by the reduced tumour size may represent a selective toxicity to proliferative cells.

DPCs are shown to induce by many carcinogens [11]. DNAs may result in large deletions during DNA replication, possibly leading to the interaction or loss of DNA sequences that may be important for tumour suppression [21]. It has been previously reported by us that DNA protein crosslinks that occurs during experimental rat hepatocarcinogenesis, was substantially reduced by chemopreventive agent vanadium [22]. *In vitro *studies by Ducore further revealed the presence of DNAs in the murine leukemia cell line L1210 that were removed in the presence of theophylline [23]. Thus fish oil-mediated reduction of DPCs clearly demonstrates the promising inhibitory effect of fish oil to an appreciable extent in mammary carcinogenesis.

Cell proliferation is a fundamental process integral to carcinogenesis. It is considered to play an important role in several steps of carcinogenic process: initiation, promotion and progression. There are multitudes of factors known to regulate cell proliferation in breast cancer. It was found by Ip *et al *that selenium substantially reduced cell proliferation during mammary preneoplasia in experimental animals [24]. Again, it was also shown by Shah *et al *that reduction of cell proliferation of breast cancer cell line SKBR3 occurred upon treatment with retinoic acid [25]. Our results of cell proliferation studies did show a significant reduction of proliferating preneoplastic cells in the mammary tissue upon fish oil supplementation.

The most common anti-apoptotic lesion that is detected in cancers is the inactivation of the p53 tumour-suppressor pathway. p53 is evolved in higher organisms to prevent tumour development. Several responses can be provoked by p53, including cell-cycle arrest, senescence, differentiation and apoptosis, with the option chosen being dependent on many factors that are both intrinsic and extrinsic to the cell. In most cases, induction of p53 leads to an irreversible inhibition of cell growth, most decisively by activating apoptosis [26]. Studies by Srivastava *et al *have shown that there was elevation of p53 expression during the inhibition of experimental mammary carcinomas by human chorionic gonadotropin (hCG) treatment [27]. It was also evidenced by Kim *et al *that chloroform extract of *Caesalpinia sappan*, increased the expression of p53 in head and neck cancer cells *in vitro *[28]. It can be mentioned that the inhibition of mammary preneoplasia may be mediated through the elevated expression of p53 gene. The decrease in the expression of p53 shows a parallel decrease in C-Myc and IL-Iβ-converting enzyme. Maxepa treatment showed an elevation in the expression of p53 in parallel with C-Myc and ICE genes when compared to carcinogen control group. This elevated expression was maintained for at least 15 days. This increase is also followed by significant elevations of C-Myc and p53 mRNAs. Though p53 is the most common mutated genes in human cancers, development of malignancies retain the wild-type p53 gene that is associated with its reduced activity due to overexpression of MdM2 and other cellular factors that down-regulate its level (29). Therefore, a substantial effort is presently focused on developing p53 functions in tumours harboring repressed wild-type p53.

## Conclusion

The involvement of fish oil (Maxepa) in suppressing neoplastic transformation of mammary tissue might be mediated through the decrease of DPCs, cell proliferation and elevation of p53 tumour suppressor protein leading to apoptosis. However, its exact molecular action in combating mammary carcinogenesis requires further study. The activation of p53 by fish oil (29) may appear therefore to be a promising effective approach for the prevention of mammary tumour development.

## Methods

### Animals and treatment

Inbred virgin female Sprague-Dawley rats obtained from Indian Institute of Chemical Biology, CSIR, (Kolkata, India) were used for the experiments. Animals of 20 ± 2 days (3 weeks) old were housed in animal cages (Tarsons) in a room, which was maintained temperature (25 ± 1°C) and humidity (50–60%) and exposed to a 12:12 hour light and dark cycle. All rats were fed a normal diet (5% mixed fat) (Ralston Purina Co., St. Louis, Mo) [6], [Table 1] and de-mineralized drinking water *ad libitum*. The rats were acclimatized to the laboratory conditions for a period of 2 weeks before the start of the experiment. The recommendations of Jadavpur University's "Institutional Animal Ethics Committee" ["Committee for the Purpose of Control and Supervision of Experiments on Animals" (CPCSEA Regn. No. 0367/01/C/CPCSEA) India] for the care and use of laboratory animals were strictly followed throughout the study.

**Table 1:** Diet Composition

**Ingredient**	**Amount**
Mixed Fat	5%
Casein (g)	17.10
DL-Methionine (g)	0.30
Dextrine (g)	45.50
Sucrose (g)	22.70
AIN-salt mixture (g)	3.50
AIN-vitamins (g)	1.00
Cellulose (g)	5.00

The calculated energy value (metabolizable energy) for the diet is 3.87 K.cal/g.

The entire experiment consisted of two parts: (I) histopathological and immunohistochemical estimations (II) morphological study of mammary tumors. For part (I), a total of 80 animals were divided into four groups, denoted as A, B, C and D. For histological and immunohistochemical studies (part I) twenty animals from each group (4 rats/cage) were used. At 7 weeks of age animals of group B and C were given a single, i.v. tail vein DMBA injection at a dose of 5 mg DMBA/2 ml corn oil/kg body weight in female Sprague-Dawley rats [30]. Since, 0.2 ml of the oil was injected; the concentration of DMBA in the corn oil was 0.5 mg DMBA/0.2 ml corn oil/100 g body weight. For the morphological study (part II), a total of 250 rats were assigned to the carcinogen control group (i.e. group B) and another 250 were assigned to the DMBA + fish oil-treated group (i.e. group C). Ten animals from each group (i.e. group B and C) were sacrificed each week starting 10 weeks (17-weeks of animal age) after the DMBA injection and continued to 35 weeks (42 weeks of animal age). The treatments for various groups were as follows:

#### Group A

Normal control animal at 7 weeks of age were given a single tail vein injection of 2 ml corn oil/kg body weight.

#### Group B

DMBA-administered animals served as the carcinogen control.

#### Group C

DMBA + fish oil [Maxepa, (Maxepa^® ^as manufactured by Merck Limited, Goa, India) supplemented at a dose of 0.5 ml ≈ 90 mg eicosapentaenoic acid (EPA) and 60 mg docosahexaenoic acid (DHA)] supplemented group. Each rat was given fish oil (Maxepa) orally, 2 weeks (5 weeks of animal age) prior to DMBA injection, via an intragastric tube according to Karmali *et al *[6].

#### Group D

Served as the fish oil (Maxepa) control.

Fish oil supplementation was started 2 weeks (5 weeks of animal age) prior to DMBA administration and continued until the termination of the experiments. The dose was given once daily, for 24 (31 weeks of animal age) weeks for histopathological and immunohistochemical analysis and 35 (42 weeks of animal age) weeks for morphological study. Fatty acid composition in the oil was determined by gas-liquid chromatography (GLC). The samples were eluted on a capillary BD23 column (J&W Scientific) after saponification with sodium hydroxide in methanol and transmethylation of the fatty acids with boron-trifluoridfe in methanol [31]. Food and liquid consumption were monitored daily by subtracting from what was provided anything not consumed by 08:00 hours the next day, from which, the approximate food and liquid consumptions were calculated per day per rat. Close monitoring of the individual rat with marking in the tail was made. The body weight also was recorded weekly throughout the experiment and as such, there was a continuous monitoring of food and water intakes of the individual rat during the course of experiment. The animals were palpated twice a week to check for the development of palpable mammary tumours and the time of tumour appearance was also recorded.

### Histological evaluation of mammary tissue

Twenty-four (31 weeks of animal age) weeks after the carcinogen or vehicle treatment, animals from each group were randomly selected; the thoracic and abdominal inguinal mammary tissues were excised from ether-anesthetized rats, fixed in 10% formalin and processed for histological studies. The tissues were dehydrated through 70, 90 and 100% alcohol and embedded in low melting-point paraffin wax. Sections of 5 μm thickness were cut and placed serially on glass slides. The sections were deparaffinized in xylene and rehydrated through 100, 90 and 70% alcohol. Three contiguous sections were made from each mammary tissue and stained with hematoxylin and eosin for histological evaluation using light microscopy. The histological slides were coded so that the particular sample identity was unknown to the individual making the assessment.

### Morphological study

Ten animals from each group were sacrificed each week starting 10 (17 weeks of animal age) weeks after the DMBA injection and continuing for up to 35 (42 weeks of animal age) weeks. The animals were anesthetized with ether and opened by a midline incision from the pubis to the submaxillary area. The skin was dissected to expose six pairs of mammary glands. Any gross modification of the mammary fat pad by vascularization or presence of any palpable as well as non-palpable tumours was recorded. Only well-developed adenocarcinomas, confirmed through histology according to the criteria of Young and Hallows [32], is reported in the Results section. Tumour size was calculated using the formula for the volume of a prolate spheroid: volume = 4/3 × 3.14 × (length/2) × (width/2) × (depth/2). The width measurement was used as the depth of the tumor [33]. The diameter measurements were further made by using a calipers and the volume of the tumor was calculated from the diameter using a stereoscopic microscope equipped with an intraocular reticule [34]. Mammary gland was exposed for the detection of non palpable tumors only at autopsy.

### Isolation of DPCs

DNA-protein complex (DPCs) from cells was isolated with slight modifications as described by Zhitkovich and Costa [11] and reported previously from our laboratory [22]. A bovine serum albumin was taken as standard blank. The DPC-coefficient was determined as a ratio of the percentage of SDS precipitable DNA in treated sample to the percentage of SDS precipitable DNA in untreated control sample. Fluorescent measurements were made using model LS45 luminescence spectrometer (Perkin Elmer, UK) with NB 360 excitation and SC 450 emission filters. For protein estimation, the isolated DPCs were nuclease-digested for 1 h at 37°C; protein was estimated according to Lowry et al. [35].

### Cell proliferation assay by immunostaining with 5'-bromo-2-deoxyuridine (BrdU)

At 6 hours before sacrifice ten animals from each group were given i.p. injections of BrdU at a dose of 50 mg/kg body weight. Only the rats in diestrus phase were used as a standard protocol. The diestrus phase was determined by histological examination of vaginal smears. The animals were anesthetized with ether and mammary tissues were dissected out. Immunohistochemical staining was done according to Zhu et al [36]. Briefly, tissue sections were exposed to 0.3% hydrogen peroxide in ethanol for 10 min to block endogenous peroxidase and treated with 2N HCl for 1 h and incubated with trypsin 0.1% for 20 min and then with normal goat serum for 20 min at room temperature. After tissue sections were incubated with the primary antibody at room temperature (anti-BrdU mouse monoclonal antibody, Sigma) in a humid chamber (1:125 for 2 h) and then with biotinylated anti-mouse IgG (1:200; Sigma) and then with streptavidin horse radish peroxidase which binds to biotin with washing in PBS after each incubation. Visualization was revealed by reaction with 3,3'diaminobenzidine (DAB; Sigma) and 0.04% hydrogen peroxide. All slides were counterstained with hematoxylin, rinsed, dehydrated and mounted with per mount. Dark brown stains identify the cells incorporating the BrdU-label. Approximately 200 cells were counted per field, 10 fields were examined per slide and 10 slides were examined per group. The labeling index (LI) was calculated as % of BrdU positive nuclei per total number of cells counted.

### Immunostaining of p53

Immunohistochemical detection of p53 protein in cold acetone fixed, paraffin embedded mammary sections was performed by the avidin-biotin-peroxidase-complex method [37]. Briefly 5 μM thin sections on lysine-coated slides were deparaffinized and rehydrated. For immunolabeling of p53, antigen retrieval was facilitated by heating the sections in citrate buffer (pH 6.0) for 20 min. Then Endogenous peroxidase activity was blocked with 1% H_2_O_2 _in 0.1 M Tris-Nacl (PH-7.6) for 30 mins. After incubation in 5% normal goat serum sections were then separately incubated overnight at 4°C with the primary antibody, anti-sheep p53 antibody (Sigma) at 1:200 dilution in 1% BSA. Sections were then incubated with a biotinylated secondary antibody goat antirabbit IgG (Sigma) for 30 mins at 37°C with 1:100 dilutions. This was followed by incubation with streptavidin peroxidase (1:100) for 1 hour and subsequent chromagen development with 3-amino 9 ethyl carbazole (AEC-H_2_O_2 _solution) (AEC-10 mg, N, N dimethyl formamide-2.5 ml, 0.1 N acetate buffer-47.5 ml, 3% H_2_O_2_-0.5 ml.) for 4–5 min. The sections were then counterstained with Harris hematoxylin, dehydrated and mounted and served as positive control. Negative controls were prepared following all the above-mentioned steps omitting the primary antibody. Approximately 200 cells were counted per field, 10 fields were examined per slide and 10 slides were examined per group.

### Statistical analysis

Comparison of the incidence of mammary tumour in different experimental groups was carried out using Fischer's exact probability test. Average standard deviation and standard error were calculated. Statistical analyses were performed using One Way ANOVA for multiple comparisons, with the level of significance set at 5%. Data were further analyzed using Student's t-test. The criteria of significance were taken as P < 0.05.

## Competing interests

**Contract Grant Sponsor**: Council of Scientific and Industrial Research (CSIR), Government of India, [Grant No. 9/96(470)2K5-EMR-I].

## Authors' contributions

Authors 1 SM, and 2 TC carried out the rat mammary carcinogenesis, morphology and histopathology. Authors 1 SM, 2 TC, 3 SD and 4 KS made cell proliferation and DPC studies. Author 5 BR confirmed the results of DPC and cell proliferation. The planning, designing and coordination of the experimental work was made by author 6 MC, the principal investigator.

## References

[B1] Wu M, Harvey KA, Ruzmetov N, Welch ZR, Sech L, Jackson K, Stillwell W, Zaloga GP, Siddiqui RA (2005). Omega-3 polyunsaturated fatty acids attenuate breast cancer growth through activation of a neutral sphingomylinase-mediated pathway. Int J Cancer.

[B2] Hong MY, Bancroft LK, Turner ND, Davidson LA, Murphy ME, Carroll RJ, Chapkin RS, Lupton JR (2005). Fish oil decreases oxidative DNA damage by enhancing apoptosis in rat colon. Nutn Cancer.

[B3] Suzuki S, Akechi T, Kobayashi M, Taniguchi K, Goto K, Sasaki S, Tsugane S, Nishiwaki Y, Miyaoka H, Uchitomi Y (2004). Daily omega-3 fatty acids intake and depression in Japanese patients with newly diagnosed lung cancer. Brit J Cancer.

[B4] Maillard V, Bougnoux P, Ferrari P, Jourdan ML, Pinault M, Lavillonniere F, Body G, Floch OL, Chajes V (2002). N-3 and N-6 fatty acids in breast adipose tissue and relative risk of breast cancer in a case-control study in Tours, France. Int J Cancer.

[B5] Hardman WE (2002). Omega-3 fatty acids to augment cancer therapy. J Nutr.

[B6] Karmali RA, Marsh J, Fuchs C (1984). Effect of omega-3 fatty acids on growth of a rat mammary tumor. J Natl Cancer Inst.

[B7] Singletary K, Liae CH (1989). Ellagic acid effects on the carcinogenicity, DNA binding and metabolism of 7, 12 dimethylbenz(α)anthracene (DMBA). In Vivo.

[B8] Iino Y, Yoshida M, Sugamata N, Maemura M, Ohwada S, Yokoe Y, Ishikita T, Horiuchi R, Morishata Y (1992). 1α-hydroxy vitamin D hypercalcemia, and growth suppression of 7, 12-dimethylbenz(α)anthracene induced rat mammary tumors. Breast Cancer Res Treat.

[B9] Witty JP, Cempka T, Coffe JRJ, Matrisian LM (1995). Decreased tumor formation in 7, 12dimethylbenzanthracene-treated stromely-sin1 transgenic mice is associated with alterations in mammary epithelial cell apoptosis. Cancer Res.

[B10] Nietert N, Kellicut LM, Kubinski H (1974). DNA protein complexes produced by a carcinogen, βproiotlactone. Cancer Res.

[B11] Zitkovitch A, Costa MA (1992). Simple sensitive assay to detect DNA protein cross-links in intact cells and in vivo. Carcinogenesis.

[B12] Ramfrex P, Razo LMD, Gutierrez-Rufz MC, Gonesebatt ME (2000). Arsenite induces DNA protein cross-links and cyutokeratin expression in the WRL 68 human hepatic cell line. Carcinogenesis.

[B13] Barret JC (1993). Mechanisms of multistep carcinogenesis and carcinogen risk assessment. Environ Health Perspect.

[B14] Freudenheim JL, Bonner M, Krishnan S, Ambrosone CB, Graham S, McCann SE, Moysich KB, Bowman E, Nemoto T, Shields GS (2004). Diet and alcohol consumption in relation to p53 mutations in breast tumors. Carcinogenesis.

[B15] Bishayee A, Oinam S, Basu M, Chatterjee M (2000). Vanadium chemoprevention of 7, 12-dimethylbenz(α)anthracene-induced rat mammary carcinogenesis: probable involvement of representative hepatic phase I and II xenobiotic metabolizing enzymes. Breast Cancer Res Treat.

[B16] Harris RE, Alshafie GA, Abous-Issa H, Siebert K (2000). Chemoprevention of breast cancer in rats by Celecoxib, a cyclooxygenase 2 inhibitor. Cancer Res.

[B17] Sinclair HM, Horrobin DF (1990). History of essential fatty acids: Omega-6 essential fatty acids: pathophysiology and roles in clinical medicine.

[B18] Nunez EA (1993). Fatty acids and cell signaling. Prostaglandins Leukotrienes and EFAs.

[B19] Woutersen RA, Appel MJ, Garderen-Hoetmer AV, Wijnands MVW (1999). Dietary fat and carcinogenesis. Mutation Res.

[B20] Welsch CW, Dettoog JV, O'Connor DH (1998). Influence of caffeine and/or coffee consumption on the initiation and promotion phases of 7, 12-dimethylbenz(α)anthracene-induced rat mammary gland tumorigenesis. Cancer Res.

[B21] Miller CA, Cohen MD, Costa M (1991). Complexing of actin and other nuclear proteins to DNA by cis-diamminedichloroplatinum(II) and chromium compounds. Carcinogenesis.

[B22] Chakraborty T, Ghosh S, Datta S, Chakraborty P, Chatterjee M (2003). Vanadium suppresses sister-chromatid exchange and DNA-protein crosslink formation and restores antioxidant status and hepatocellular architecture during 2-acetylaminofluorene-induced experimental rat hepatocarcinogenesis. J Exp Ther Oncol.

[B23] Ducore JM cis-Diamminedichloroplatinum(II) (DDP)-induced crosslinking and crosslink removal in L1210 cells in vitro after theophylline co-treatment. Biochem Pharmacol.

[B24] Ip C, Thompson HJ, Ganther HE (2000). Selenium Modulation of Cell Proliferation and Cell Cycle Biomarkers in Normal and Premalignant Cells of the Rat Mammary Gland. Cancer Epidemiol Biomarkers Prev.

[B25] Shah S, Michael JP, Easwaran V, Brown PH, Byers SW (2000). The Role of Cadherin, β-Catenin, and AP-1 in Retinoid-regulated Carcinoma Cell Differentiation and Proliferation. J Biol Chem.

[B26] Vousden KH, Lu X (2000). Live or let die: the cell's response to p53. Nature Rev on Cancer.

[B27] Srivastava P, Russo J, Russo IH (1997). Chorionic gonadotropin inhibits rat mammary carcinogenesis through activation of programmed cell death. Carcinogenesis.

[B28] Kim EC, Hwang YS, Lee SK, Park MH, Jeon BH, Jeon CD, Lee SK, Yu HH, You YO (2005). Caesalpinia sappan induces cell death by increasing the expression of p53 and p21WAF1 in head and neck cancer cells. Am J Clin Med.

[B29] Tabakin-Fix Y, Azran I, Schavinky-Khrapunsky Y, Levy O, Aboud M (2006). Functional inactivation of p53 by human T-cell leukemia virus type 1 Tax protein: mechanisms and clinical implications. Carcinogenesis.

[B30] Oinam S, Karmakar R, Roy A, Indira BN, Chatterjee M (1999). Effect of vitamin D_3 _on carcinogen-modified liver enzymes and tumor incidence in experimental rat mammary carcinogenesis. Eur J Cancer Prev.

[B31] Appel MJ, Woutersen RA (1996). Dietary fish oil (MaxEPA) enhances pancreatic carcinogenesis in azaserine-treated rats. Brit J Cancer.

[B32] Young S, Hallowes RC, Turusov VS (1973). Tumor of the mammary gland: Pathology of tumors in Laboratory Animals. International Agency for Research on Cancer.

[B33] Hardman WE, Moyer MP, Cameron IL (1999). Fish oil supplementation enhanced CPT-11 (irinotecan) efficacy against MCF7 breast carcinoma xenografts and ameliorated intestinal side-effects. Brit J Cancer.

[B34] Hubbard NE, Lim D, Erickson KL (1998). Alteration of murine mammary tumorigenesis by dietary enriched with n-3 fatty acids in fish oil. Cancer Lett.

[B35] Lowry OH, Rosebrought NJ, Farr AL, Randall RJ (1951). Protein measurement with the folin phenol reagent. J Biol Chem.

[B36] Zhu Z, Jiang W, Thompson HJ (1991). Effect of energy restriction on the expression of cyclin D1 and P27 during premalignant and malignant stages of chemically induced mammary carcinogenesis. Mol Carcinog.

[B37] Jin R, Chow VTK, Tan PH, Dheen ST, Duan W, Bay BH (2002). Metallothionein 2A expression is associated with cell proliferation in breast cancer. Carcinogenesis.

